# Protein Homeostasis in Amyotrophic Lateral Sclerosis: Therapeutic Opportunities?

**DOI:** 10.3389/fnmol.2017.00123

**Published:** 2017-05-02

**Authors:** Christopher P. Webster, Emma F. Smith, Pamela J. Shaw, Kurt J. De Vos

**Affiliations:** Sheffield Institute for Translational Neuroscience (SITraN), Department of Neuroscience, University of SheffieldSheffield, UK

**Keywords:** protein homeostasis, protein aggregation, amyotrophic lateral sclerosis (ALS), motor neuron disease, autophagy, chaperonins, unfolded protein response (UPR), proteostasis

## Abstract

Protein homeostasis (proteostasis), the correct balance between production and degradation of proteins, is essential for the health and survival of cells. Proteostasis requires an intricate network of protein quality control pathways (the proteostasis network) that work to prevent protein aggregation and maintain proteome health throughout the lifespan of the cell. Collapse of proteostasis has been implicated in the etiology of a number of neurodegenerative diseases, including amyotrophic lateral sclerosis (ALS), the most common adult onset motor neuron disorder. Here, we review the evidence linking dysfunctional proteostasis to the etiology of ALS and discuss how ALS-associated insults affect the proteostasis network. Finally, we discuss the potential therapeutic benefit of proteostasis network modulation in ALS.

## Introduction

**FIGURE 1:**
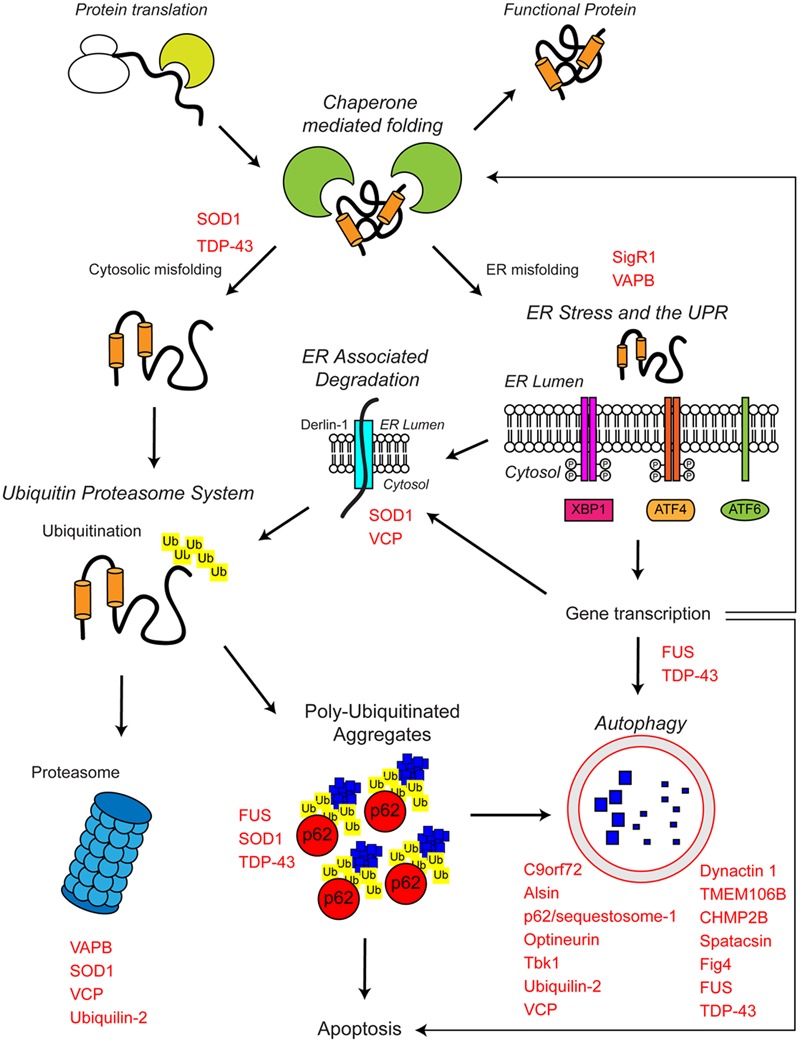
**The proteostasis network and ALS.** Protein folding occurs co-translationally at the ribosome with the aid of molecular chaperones, including Hsp70. Correct folding is essential for protein function. Protein folding and refolding continues in the cytosol and the endoplasmic reticulum (ER) lumen. Chronic misfolding in the cytosol leads to targeting of misfolded substrates to the ubiquitin proteasome system (UPS). Poly-ubiquitin chains target substrates for degradation by the proteasome. Overwhelming of the UPS can lead to poly-ubiquitinated aggregate formation, which are cleared by the autophagosome–lysosome pathway. Chronic misfolding in the ER leads to the induction of ER stress and activation of the unfolded protein response (UPR). The UPR leads to altered gene transcription, upregulating ER associated degradation (ERAD) and autophagy. The proteostasis network seeks to restore protein homeostasis, but failure of the pathway leads to the aggregation of potentially toxic species. Disruption of the proteostasis network is prevalent in the pathogenesis of ALS. A large number of ALS-associated genes (indicated in red) directly or indirectly regulate the proteostasis network. In addition, some ALS-associated proteins such as FUS, TDP-43, and SOD1 are also substrates of these pathways. For further details please refer to the main text.

The proteostasis network is a complex regulatory network that maintains proteostasis. The proteostasis network consists of several pathways that control protein biosynthesis, folding, trafficking, and clearance (degradation) and responds to specific protein stress pathways such as the unfolded protein response (UPR) in the endoplasmic reticulum (ER), the mitochondrial UPR and the cytosolic heat shock response (**Figure 1**).

Proteins are constantly turned over to ensure a steady supply of functional proteins. Newly synthesized proteins fold into their specific three-dimensional shape co-translationally as the nascent polypeptide chain emerges from the ribosome. The specific three-dimensional structure of a protein, which is in part determined by its amino acid sequence, is crucial to its function. A number cytosolic and ER resident folding factors aid the complex process of protein folding, such as chaperones and co-chaperones of the heat shock protein (Hsp) family, peptidyl prolyl *cis/trans* isomerases, and oxidoreductases (reviewed in Braakman and Bulleid, 2011; Kim et al., 2013). Nevertheless, the nature of protein folding and the cellular environment is such that unfolding and misfolding are relatively common events. Upon protein misfolding, specific protein stress pathways such as the heat shock response and UPR are activated to boost chaperone levels and aid refolding or to stimulate removal of terminally misfolded proteins to prevent protein aggregation and proteotoxic stress.

Eukaryotic cells have two major pathways of protein degradation: the proteasome and the lysosome. The proteasome is a multimeric ATP-dependent protease complex that selectively recognizes ubiquitinated substrates. Degradation by the proteasome requires protein unfolding and relies on chaperones to prevent proteins from aggregating (reviewed in Hershko and Ciechanover, 1998). Autophagy denotes the delivery of cytoplasmic components to the lysosome. Autophagy can be separated into three types depending on how the substrate is delivered to the lysosome: chaperone mediated autophagy (CMA), microautophagy and macroautophagy, herein termed autophagy (reviewed in Bento et al., 2016). Aggregated proteins are mostly removed by autophagy, a process that is also called aggrephagy (reviewed in Lamark and Johansen, 2012).

Ultimately proteostasis collapse due to failure of the proteostasis network to refold, degrade or effectively sequester and compartmentalize aggregation-prone, misfolded and potentially toxic protein species is deleterious to cells. Neuronal cells appear to be particularly vulnerable to disturbances in proteostasis because they are long-lived post-mitotic cells that are not able to dilute out protein aggregates during cell divisions (Son et al., 2012). Furthermore, as the ability of cells to maintain proteostasis declines with aging it is not surprising that aberrant protein folding and aggregate deposition in neurons is a common feature of age-associated neurodegenerative disease.

Here, we review the evidence linking dysfunctional proteostasis to the etiology of amyotrophic lateral sclerosis (ALS) and discuss how ALS-associated insults affect the proteostasis network.

## Loss of Protein Homeostasis in ALS

ALS is a progressive adult onset motor neuron disease characterized by selective degeneration of upper and lower motor neurons in the motor cortex, brainstem, and spinal cord. The progressive deterioration of the motor system leads to muscle wasting, paralysis and eventual premature death, most commonly due to respiratory failure. Death occurs on average within approximately 3 years of symptom onset (reviewed in Kiernan et al., 2011). The causes of ALS are numerous and complex, but remain incompletely understood. Proposed mechanisms include, among others, oxidative stress, mitochondrial dysfunction, defective axonal transport, RNA toxicity, excitotoxicity, neuroinflammation, and loss of protein homeostasis (reviewed in Ferraiuolo et al., 2011; De Vos and Hafezparast, 2017). While most ALS cases have no clear genetic basis (sporadic ALS), approximately 10% of ALS is inherited, usually in an autosomal dominant fashion (familial ALS). Studies of familial ALS cases have revealed multiple ALS-associated genes. While these genes play significant roles in a range of essential cellular processes including RNA processing, mitochondrial function and endosomal trafficking, a significant number of these have been linked to different aspects of the proteostasis network (**Table 1** and **Figure 1**).

**Table 1:** Potential impact of ALS-associated genes on protein quality control.

ALS locus	Gene	Protein	Potential consequence of mutation on protein homeostasis
*ALS1*	*SOD1*	Superoxide dismutase 1	Aggregate formation leading to depletion of available chaperones, induction of ER stress due to impairment of UPR and ERAD, impaired UPS and autophagy
*ALS2*	*ALS2*	Alsin	Reduced Vps34 activation and autophagosome formation
*ALS5*	*SPG11*	Spatacsin	Reduced autophagosome–lysosome reformation
*ALS6*	*FUS*	RNA-binding protein FUS	Aggregate formation, reduced autophagy related gene expression and impaired substrate delivery to autophagosome
*ALS8*	*VAPB*	Vesicle-associated membrane protein-associated protein B	Defective UPR activation and increased ER stress
*ALS10*	*TARDBP*	TAR DNA-binding protein 43	Redistribution of TDP-43 to cytoplasmic aggregates, reduced autophagy related gene expression and defective substrate delivery to autophagosomes
*ALS11*	*FIG4*	Fig4	Reduced autophagosome clearance
*ALS12*	*OPTN*	Optineurin	Impaired autophagy substrate recruitment to autophagosomes
*ALS14*	*VCP*	Valosin-containing protein	Impaired mitochondrial proteostasis and ERAD, defective UPS degradation, and reduced autophagosome maturation
*ALS15*	*UBQLN2*	Ubiquilin-2	Defective substrate delivery to the proteasome and proteasomal clearance. Impaired substrate delivery to autophagosomes
*ALS16*	*SIGMAR1*	Sigma non-opioid intracellular receptor 1	Dysfunctional ER–mitochondria communication, calcium dysfunction, and ER stress
*ALS17*	*CHMP2B*	Charged multivesicular body protein 2B	Impaired endocytic trafficking, autophagosome–lysosome fusion and reduced autophagic clearance
*ALS-FTD1*	*C9orf72*	C9orf72	Reduced functional protein, defective Rab-mediated trafficking and impaired autophagy induction. Additional formation of DPR proteins from expanded repeat
*ALS*	*DCTN1*	Dynactin 1 (p150, glued homolog, *Drosophila*)	Altered axonal transport and vesicle trafficking, impaired signaling endosome trafficking and reduced autophagosome transport
*ALS*	*SQSTM1*	p62/sequestosome-1	Impaired autophagy substrate recruitment to autophagosomes
*ALS*	*TBK1*	TANK binding kinase-1	Reduced phosphorylation of autophagy receptors, reducing ubiquitin and LC3-II binding capacity

*Pathogenic variants of the proteins in this table have been linked to disrupted protein homeostasis (ref: http://alsod.iop.kcl.ac.uk/home.aspx; Abel et al., 2012). DPR, dipeptide repeat; ER, endoplasmic reticulum; ERAD, endoplasmic reticulum associated degradation; LC3, microtubule-associated protein 1 light chain 3; UPR, unfolded protein response; UPS, ubiquitin proteasome system.*

There is a significant clinical, neuropathological, and genetic overlap between ALS and frontotemporal dementia (FTD), a common form of early onset dementia that is characterized by changes in behavior and personality or language dysfunction. Up to 15% of ALS cases are clinically diagnosed with FTD and approximately 50% of FTD cases display motor symptoms (reviewed in Swinnen and Robberecht, 2014).

### Neuropathological Evidence for Dysfunctional Proteostasis in ALS

Intracellular proteinaceous inclusions are a hallmark neuropathological feature of ALS. Inclusions are found in both degenerating neurons and surrounding glia (Piao et al., 2003; Nishihira et al., 2008; Zhang et al., 2008) and are found not only in the brainstem and spinal cord, but also in the cerebellum, hippocampus, and the frontal and temporal lobes (reviewed in Al-Chalabi et al., 2012). The most common inclusions are of ubiquitinated proteins, which are found in both the upper and lower motor neurons (Neumann et al., 2006), and are suggestive of defects in protein turnover (Blokhuis et al., 2013). Based on their morphology, these ubiquitinated inclusions are subdivided into skein-like inclusions that are filamentous in structure, and rounded Lewy body-like inclusions (Leigh et al., 1988; Lowe et al., 1988; Kato et al., 1989). ALS-associated ubiquitinated inclusions are typically positive for p62/sequestosome-1 (King et al., 2011), a ubiquitin binding protein involved in autophagy (see below). In the vast majority of sporadic and familial ALS cases ubiquitinated protein inclusions are positive for Tar DNA-binding protein of 43 kDa (TDP-43) (Arai et al., 2006; Neumann et al., 2006). TDP-43 was also identified as the pathological protein in frontotemporal lobar degeneration (FTLD)-related ubiquitinated inclusions, supporting the idea that ALS and FTD reside on the same spectrum of disease (Arai et al., 2006; Neumann et al., 2006; reviewed in Swinnen and Robberecht, 2014). Not only are aggregates of wild type TDP-43 found in nearly all cases of disease, but mutations in the *TARDPB* gene that encodes TDP-43 are also causative for ALS (Kabashi et al., 2008; Sreedharan et al., 2008). ALS-associated mutations in *TARDBP* lead to cytoplasmic TDP-43 mislocalisation and its aberrant incorporation into neurotoxic ubquitinated cytoplasmic aggregates (Barmada et al., 2010). Inhibition of the proteasome or autophagy leads to the aggregation of TDP-43 (Urushitani et al., 2010; Wang et al., 2010) (see below).

Although the majority of ubiquitinated inclusions are immunoreactive for TDP-43, notable exceptions are the inclusions found in ALS patients with mutations in *SOD1* or *FUS*, which are negative for ubiquitinated TDP-43 but immunoreactive for mutant aggregated Cu/Zn superoxide dismutase (SOD1) and fused in sarcoma protein (FUS) respectively (Watanabe et al., 2001; Wang et al., 2002; Mackenzie et al., 2007; Kwiatkowski et al., 2009; Vance et al., 2009). Similar to TDP-43, mutant FUS demonstrates abnormal cytoplasmic redistribution and aggregation (Kwiatkowski et al., 2009; Vance et al., 2009; Dormann et al., 2010). Further familial ALS-associated mutant proteins that are prone to aggregation are valosin containing protein (VCP), dynactin-1 (DCTN1), optineurin (OPTN) and ubiquilin-2 (UBQLN2) (Levy et al., 2006; Maruyama et al., 2010; Deng et al., 2011; Koppers et al., 2012).

C9orf72-related ALS presents an outlier to classical ALS pathology. In C9orf72-related ALS TDP-43 proteinopathy is present, but additional inclusions are p62/sequestosome-1 and ubiquitin positive, yet devoid of TDP-43 (Al-Sarraj et al., 2011; Cooper-Knock et al., 2012; Mackenzie et al., 2014). In addition, the GGGGCC repeat expansion in the *C9ORF72* gene gives rise to five species of dipeptide protein (DPR) inclusions (GA, GR, GP, PR, and PA) by repeat-associated non-AUG translation (Mann et al., 2013; Mori et al., 2013; Mackenzie et al., 2014).

Other ALS-associated inclusions include Bunina bodies (found post-mortem in approximately 86% of sporadic ALS patients) and hyaline conglomerate inclusions (Bunina, 1962; Kato et al., 1989; Murayama et al., 1989). Bunina bodies are comprised of cystatin C, transferrin, peripherin, and sortilin-related receptor CNS expressed 2 (SORCS2) and are found in the surviving lower motor neurons within the brain stem and spinal cord (Okamoto et al., 1993; Piao et al., 2003; Mizuno et al., 2006, 2011; Mori et al., 2015). They also contain small organelle fragments such as vesicles and ER (Okamoto et al., 2008; Kimura et al., 2014). Bunina bodies and TDP-43 positive inclusions have been shown to co-localize, and TDP-43 inclusion prevalence increased with Bunina body presence suggesting a synergy between the two inclusion types (Mori et al., 2010, 2014). Hyaline conglomerate inclusions are comprised of phosphorylated and non-phosphorylated neurofilaments (Hirano et al., 1984; Munoz et al., 1988; Hays et al., 2006) and their formation is possibly linked to defective axonal transport (Munoz et al., 1988; Ackerley et al., 2000, 2003).

The typical occurrence of these protein aggregates in ALS patients strongly suggest a collapse of proteostasis in ALS. These proteinaceous inclusions are replicated in a number ALS-associated animal models, including mutant SOD1, FUS, and TDP-43 transgenic mice. The fact that these models are able to effectively replicate some of the most prevalent neuropathological features of ALS suggests that the proteinaceous inclusions and aggregates are major contributors to disease pathogenesis.

### Genetic Evidence for Dysfunctional Proteostasis in ALS

As mentioned above, about 10% of ALS cases are inherited, usually in an autosomal dominant fashion (reviewed in Renton et al., 2014). A number of familial ALS-associated proteins are known to be involved in the proteostasis network, including C9orf72, VCP, p62/sequestosome-1, ubiquilin-2, optineurin, dynactin, and TANK binding kinase 1 (TBK1) (**Table 1** and **Figure 1**). The role of these genes in proteostasis is discussed in detail in the following sections.

### Altered Chaperone Function in ALS

**FIGURE 2:**
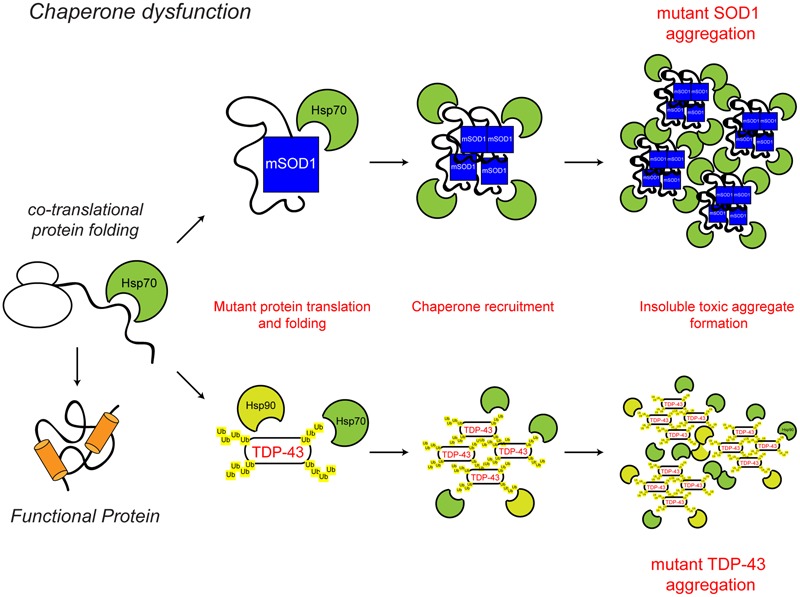
**Chaperone dysfunction in ALS.** Protein folding occurs co-translationally at the ribosome. Correct folding and re-folding continues in the cytoplasm or ER with the help of molecular chaperones and leads to correctly folded, fully functional proteins. Chaperone dysfunction has been implicated in ALS. Aggregating ALS mutant SOD1 and mislocalised TDP-43 interact with chaperones of the heat shock protein family, namely Hsp70 and Hsp90. While the recruitment of chaperones to the aggregates is likely a protective mechanism, their sequestration potentially depletes the levels of available chaperones, decreasing chaperone folding activity, therefore leading to toxicity. The ALS-associated protein aggregates and other ALS-associated defects to the chaperone system are indicated in red. For further details please refer to the main text.

Molecular chaperones assist protein folding and help maintain proteins in their native folded state. In addition, they function in proteostasis to facilitate protein unfolding and disaggregation, and the targeting of terminally misfolded proteins for degradation. In relation to neurodegeneration, perhaps the key function of chaperones is to prevent protein aggregation under conditions of stress. Altered chaperone function has been implicated in ALS (**Figure 2**).

The presence of chaperones, such as heat shock cognate protein of 70 kDa (Hsc70) and heat shock protein of 90 kDa (Hsp90) in detergent-insoluble neuronal aggregates in the SOD1G93A transgenic mouse model of familial ALS and post-mortem human sporadic ALS cases indicates that altered chaperone function may contribute to disease pathogenesis (Watanabe et al., 2001; Basso et al., 2009; Bergemalm et al., 2010). In cell based assays and mouse models, ALS mutant SOD1G93A and G85R show increased interaction with a number of chaperones, including Hsp70, suggesting that ALS-associated mutant species may result in a depletion of available chaperones and chaperone activity, therefore leading to cellular toxicity (Tummala et al., 2005; Ganesan et al., 2007). Furthermore, reduced levels of the chaperone alpha-B-crystallin (CRYAB) and increased incorporation of other molecular chaperones, including Hsc70, into the insoluble aggregate fraction are features of a faster progressing phenotype in SOD1G93A transgenic mice (Marino et al., 2015).

As discussed above, redistribution of wild type or mutant TDP-43 to the cytoplasm and its aberrant inclusion into ubiquitinated cytoplasmic aggregates is a hallmark of ALS (Arai et al., 2006; Neumann et al., 2006). Interestingly, TDP-43 aggregates have been shown to interact with Hsp70 and Hsp90 under conditions of heat shock stress or reactive oxygen species (ROS) insult (Chang et al., 2013; Udan-Johns et al., 2014; Budini et al., 2015). The finding that activating Hsp70 reduces the insoluble aggregates of TDP-43, suggests that chaperone dysregulation could be a contributing factor to TDP-43 aggregate formation and toxicity (Chang et al., 2013). In agreement with this, knock-down of the molecular chaperones Hsp70 and Hsp90, leads to increased aggregation of TDP-43 (Zhang et al., 2010), while increasing levels of the small heat shock protein B8 (HspB8) increased the solubility of mutant TDP-43 and reduced toxicity (Crippa et al., 2010). Similarly, upregulation of the small heat shock protein CG14207 was able to reduce neurotoxicity of full length TDP-43 and the C-terminal fragment of TDP-43 in a *Drosophila* model of ALS (Gregory et al., 2012). Overexpression of Hsc70 in *Drosophila* also prevents the aggregation of FUS protein into insoluble fractions (Miguel et al., 2012).

Thus, increased chaperone levels may provide a protective mechanism in ALS. The reported upregulation of HspB1 and HspB8 chaperone expression in the lumbar spinal cord of 39 ALS cases (4 of which were familial ALS cases) compared to 19 control samples may be an indication of such a protective response (Anagnostou et al., 2010).

### ER Stress, the UPR and ALS

**FIGURE 3:**
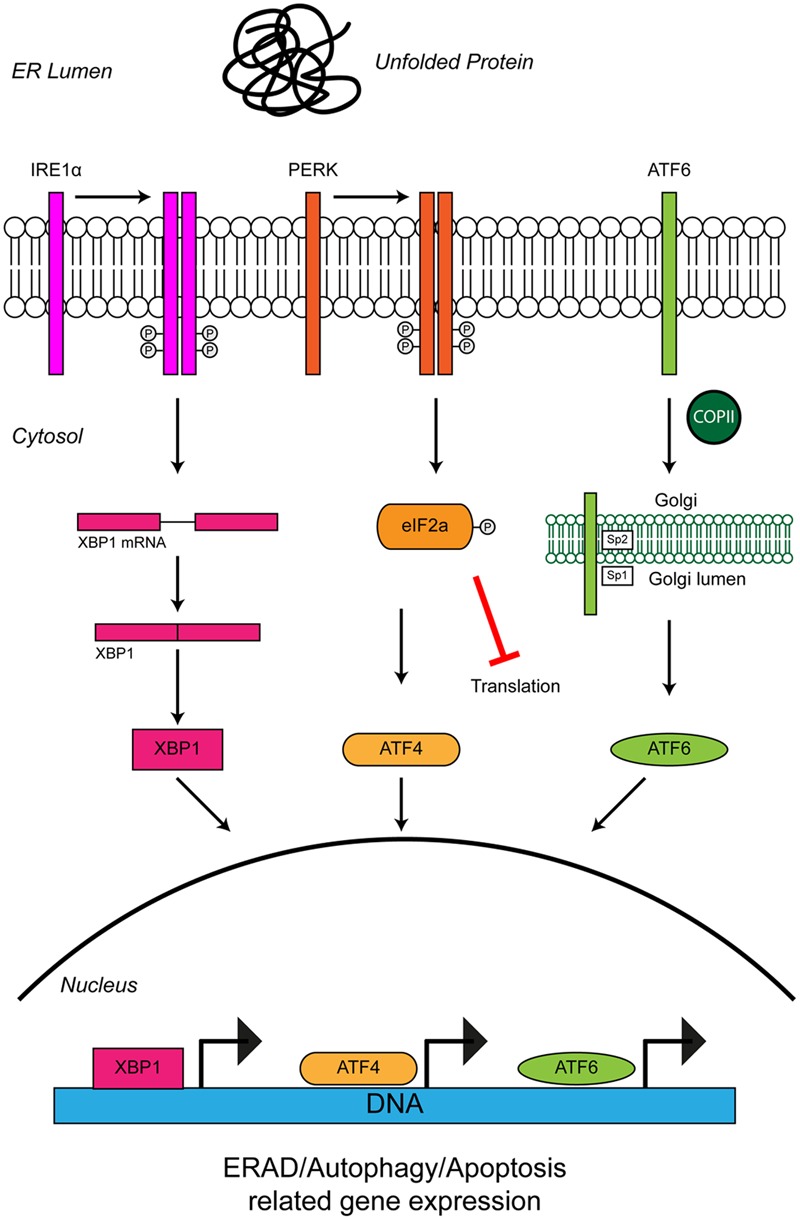
**Unfolded protein response in the ER leads to ER stress.** The accumulation of protein aggregates is sensed by three ER-stress transducers: IRE1α, PERK, and ATF6. ER stress causes IRE1α dimerisation, which activates its intrinsic RNAse activity and leads to alternative splicing of XBP1 mRNA. Spliced XBP1 forms a functional transcription factor. XBP1 increases expression of chaperone related genes and those involved in ERAD. PERK also dimerises due to ER stress. PERK dimerization leads to phosphorylation of the eukaryotic initiation factor eIF2α, thus inhibiting general protein synthesis. Inhibition of protein synthesis allows the translation of stress response transcription factor, ATF4. ATF4 increases expression of genes related to autophagy and apoptosis. Via the action of coat protein complex II (COPII), ATF6 translocates from the ER membrane to the Golgi during ER stress where it is processed by the Site 1 (Sp1) and Site 2 (Sp2) proteases. Cleavage produces a functional cytosolic fragment of ATF6. The ATF6 transcription factor induces expression of genes related to ERAD, but also XBP1, thereby promoting UPR. Chronic ER stress and UPR activation indicates the cell has failed to respond to ER stress. Under such conditions all three ER stress transducers lead to the increased expression of CHOP, which promotes apoptosis. For further details please refer to the main text.

Cells use stress sensors and inducible pathways to respond to a loss of proteostatic control. One such pathway is the UPR, an adaptive response to the accumulation of misfolded proteins in the lumen of the ER (i.e., ER stress) (**Figure 3**). ER stress induced by accumulation of unfolded proteins activates three UPR pathways mediated by three ER membrane resident stress sensors, protein kinase RNA-like ER kinase (PERK), inositol-requiring protein 1 (IRE1) and activating transcription factor 6 (ATF6) (reviewed in Hetz, 2012). The UPR tries to restore proteostasis by (i) attenuation of translation, (ii) induction of chaperones to aid protein folding, and (iii) upregulation of degradation pathways such as ER-associated degradation (ERAD) and autophagy to remove misfolded proteins. If ER stress is short-lived, the UPR restores proteostasis and the cell survives. If in contrast ER stress persists, as for example in ALS, the UPR triggers apoptosis and the cell is lost.

**FIGURE 4:**
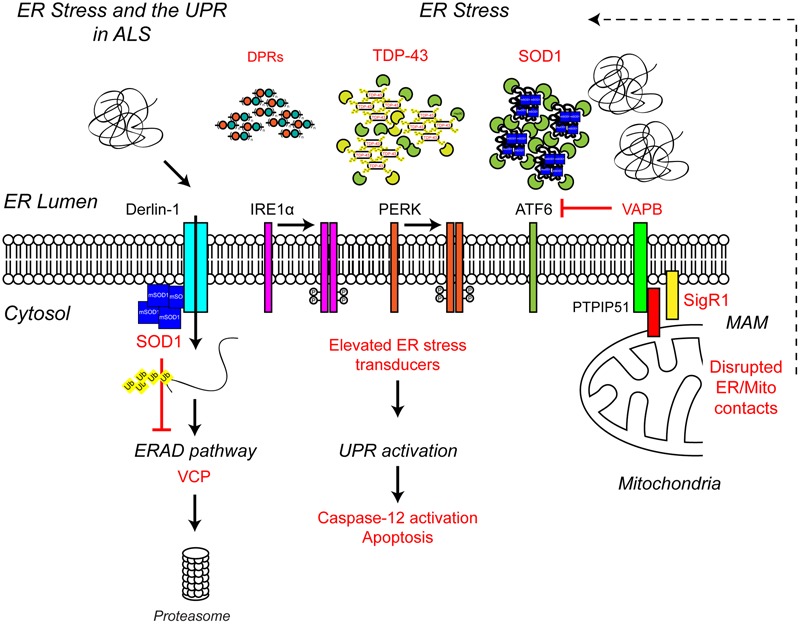
**Endoplasmic reticulum stress the UPR and ALS.** ALS-associated genes have been implicated in ER stress and the UPR. The ALS-associated genes and their positions within the UPR pathway are indicated in red, as are other ALS-associated defects to the UPR. Briefly, ALS-associated protein aggregates, including C9orf72-related DPR proteins, TDP-43 and SOD1, are sensed by the ER-stress transducers leading to chronic activation of the UPR, caspase-12 cleavage and apoptosis, while disruption of ER/mitochondria contact sites leads to dysfunctional calcium homeostasis and, in turn, elevated ER stress. Finally, mutant SOD1 aggregates interact with Derlin-1, a member of the ERAD pathway, and disrupts the proteasome-dependent degradation of misfolded ER proteins, thus promoting further ER stress. For further details please refer to the main text.

Multiple lines of evidence from ALS patients and models suggest that ER stress may be a contributing factor to the development of ALS (**Figure 4**). Amorphous, granular material indicative of unfolded proteins has been shown to accumulate in the ER of motor neurons of sporadic ALS patients (Sasaki, 2010). In line with ER stress and induction of the UPR, the ER chaperone binding immunoglobulin protein/glucose-regulated protein 78 (BiP/GRP-78) was upregulated in motor neurons of sporadic ALS patients (Sasaki, 2010) and the levels of all three UPR stress transducers were found to be elevated in cerebrospinal fluid (CSF) and spinal cord of sporadic ALS patients (Atkin et al., 2008). In addition, a number of UPR-related chaperones, including protein disulfide isomerase (PDI) were present in CSF and throughout the motor neurons of sporadic ALS patients (Atkin et al., 2006, 2008). PDI has also been found in association with SOD1-inclusions in SOD1G93A transgenic mice, and it has been suggested that PDI is involved in the removal of these aggregates (Atkin et al., 2006). In SOD1G93A transgenic mice elevated UPR stress sensor levels have been observed prior to disease onset, as early as postnatal day 5, suggesting ER stress may be an early pathogenic event in the development of ALS (Atkin et al., 2008; Saxena et al., 2009). *In vitro*, expression of mutant SOD1A4V in the NSC-34 motor neuron-like cell line induces ER stress and apoptosis (Walker et al., 2010).

Interestingly, exposure of primary motor neurons to the CSF of sporadic ALS patients leads to ER stress, activation of the UPR and neuronal degeneration (Vijayalakshmi et al., 2009, 2011). How CSF elicits ER stress and degeneration is not clear but the process appears to involve activation of Caspase-12. Caspase-12 is known to mediate ER stress-induced apoptosis, and is activated by the UPR (Nakagawa et al., 2000; Martinez et al., 2010).

Chemical induction of ER stress in NSC-34 cells led to increased TDP-43 cleavage (Suzuki et al., 2011), while overexpression of ALS-associated TDP-43A315T and Q331K mutants in Neuro2a cells led to ER stress and activation of a number of UPR stress response pathways, including increased CHOP protein levels, increased nuclear XBP1 and increased activation of ATF6 (Walker et al., 2013). This activation of the UPR by mutant TDP-43 could further promote the cytoplasmic mislocalisation of TDP-43, and therefore toxicity (Walker et al., 2013). Similarly, expression of ALS mutant FUS and C9orf72-associated poly-GA DPRs have been shown to induce ER stress in NSC-34 cells and primary neurons, respectively (Farg et al., 2012; Zhang Y.J. et al., 2014).

Several ALS-associated proteins appear to directly interfere with pathways required for proteostasis and/or UPR. ALS mutant SOD1 has been shown to interact with Derlin-1, an ER protein essential for ERAD, and perturb the ERAD pathway (Nishitoh et al., 2008). Dysfunctional ERAD induces ER stress which eventually triggers apoptosis via the ASK1 pathway (Nishitoh et al., 2008). Mutations in vesicle-associated membrane protein-associated protein B (VAPB), an integral ER protein that has been implicated in UPR activation via the IRE1 and ATF6 pathways cause ALS8 (Nishimura et al., 2004; Kanekura et al., 2006; Gkogkas et al., 2008; Suzuki et al., 2009). Overexpression of ALS mutant VAPBP56S has been shown to induce ER stress (Suzuki et al., 2009). However, VAPB expression is down-regulated in ALS8 patient-derived iPSC neurons due to reduced expression of the VAPBP56S mutant (Mitne-Neto et al., 2011), therefore it is more likely ALS-associated loss of function of VAPB may predispose motor neurons to ER stress (Kanekura et al., 2006; Gkogkas et al., 2008; Suzuki et al., 2009). Indeed, since knock-down of VAPB inhibits activation of the IRE/XBP1 pathway in response to chemical ER stressors, VAPB appears to promote the UPR in reaction to ER stress under physiological conditions (Kanekura et al., 2006). How VAPB regulates the UPR is not yet clear but may involve its role in maintaining ER/mitochondria contacts via interaction with the mitochondrial outer membrane protein PTPIP51 (De Vos et al., 2012).

ER/mitochondria contacts allow mitochondria and ER to communicate directly with each other via the exchange of calcium signals (reviewed in Paillusson et al., 2016). Under physiological conditions, mitochondrial calcium activates the rate-limiting enzymes of the Krebs cycle and thereby increases oxidative phosphorylation and ATP synthesis to match local energy demand. In turn, energized mitochondria influence ER calcium homeostasis and redox dependent ER processes such as oxidative protein folding (reviewed in Walczak et al., 2012). Disruption of ER/mitochondria contacts has been shown to induce ER stress and the UPR (Simmen et al., 2005), possibly by disturbing the variety of ER chaperones, such as BiP, calnexin, calreticulin, ERp44, ERp57, and Sigma non-opioid intracellular receptor 1 (Sig1R) that are present in mitochondria-associated ER membranes (MAMs) (Hayashi and Su, 2007). Reduced levels of VAPB have been reported in the spinal cord of sporadic ALS cases, suggesting that ER/mitochondria contacts and the UPR may be impaired as a consequence (Anagnostou et al., 2010). Consistent with this possibility, neuronal overexpression of wild type human VAPB has been shown to slow disease and increase survival in SOD1G93A transgenic mice (Kim et al., 2016) but whether this is related to the restoration of the UPR and ER/mitochondria contacts remains to be determined. Interestingly disruption of ER/mitochondria contacts appears to be a common phenomenon in ALS with reduced ER/mitochondria contact sites found in mutant SOD1, Sig1R, TDP-43, and FUS-related ALS (Lautenschlager et al., 2013; Stoica et al., 2014, 2016; Watanabe et al., 2016).

Mutations in the *SIGMAR1* gene that encodes Sig1R cause a juvenile form of ALS (ALS16) (Al-Saif et al., 2011). Sig1R1 is an ER protein that resides at ER/mitochondria contacts where it interacts with BiP and regulates calcium exchange by acting as a ligand-operated receptor chaperone for the inositol 1,4,5-trisphosphate receptor 3 (IP3R3) (Hayashi and Su, 2007). ALS-associated loss of function of Sig1R has been linked to dysfunctional ER/mitochondria communication calcium dysfunction and ER stress (Prause et al., 2013; Vollrath et al., 2014).

Mutations in the ATPase VCP cause of 1–2% of familial ALS cases as well as inclusion body myopathy (IBM) with Paget’s disease (PDB) and FTD (Johnson et al., 2010; Koppers et al., 2012). Among other functions, VCP is involved in protein degradation, ERAD, ER stress and autophagy (reviewed in Meyer and Weihl, 2014), and pathogenic mutations have been shown to impair mitochondrial proteostasis, attenuate ERAD and lead to an impaired stress response (Fang et al., 2015; Wang et al., 2016). Mutant VCP has been linked to altered TDP-43 metabolism in *Drosophila* (Ritson et al., 2010) and spinal cord motor neurons of mutant VCP transgenic mice exhibit TDP-43 pathology (Custer et al., 2010). Mutant VCP expression also leads to redistribution of wild type TDP-43 from the nucleus to the cytoplasm *in vitro* and *in vivo* (Gitcho et al., 2009; Custer et al., 2010; Ritson et al., 2010). Thus, the typical TDP-43 pathology observed in ALS may be a direct consequence of dysfunctional proteostasis.

### Proteasome Dysfunction and ALS

**FIGURE 5:**
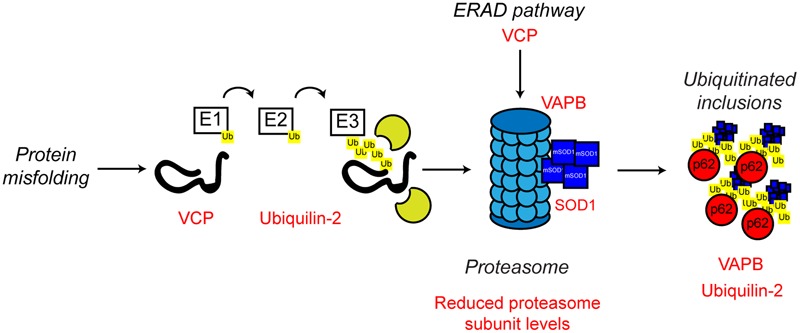
**Proteasome dysfunction in ALS.** The ubiquitin proteasome system is responsible for the degradation of poly-ubiquitinated protein substrates. Misfolded proteins are poly-ubiquitinated by the action of the E1, E2, and E3 ubiquitin ligases. Proteasome dysfunction has been implicated in ALS. Altered substrate delivery to the proteasome, mutant protein interaction with the proteasome, as in the case of mutant SOD1, and reduced proteasome function have all been implicated in ALS pathogenesis, ultimately leading to poly-ubiquitinated protein aggregate formation. The ALS-associated genes and their positions in the UPS are indicated in red, as are other ALS-associated defects. Interestingly, not only can mutant SOD1 interact with the 19S subunit of the proteasome, but mutant SOD1 is also a substrate for proteasome clearance. For further details please refer to the main text.

The characteristic pathological protein accumulations seen in ALS are indicative of defective protein clearance. In eukaryotes, the majority of misfolded proteins are degraded by the UPS in which poly-ubiquitin-tagged substrates are directed to and degraded by the 26S proteasome, a multimeric ATP-dependent protease complex comprised of the core 20S protease capped with two 19S regulatory subunits (reviewed in Eytan et al., 1989; Driscoll and Goldberg, 1990; Hoffman et al., 1992; Voges et al., 1999; Ciechanover and Kwon, 2015). Evidence from both familial and sporadic cases of ALS indicates proteasome dysfunction may be involved in disease pathogenesis (**Figure 5**).

ALS mutant SOD1 has been shown to directly interact with the 19S regulatory subunits of the proteasome, which could contribute to the observed proteasomal inhibition seen in multiple models of ALS (Urushitani et al., 2002; Kabashi et al., 2004; Cheroni et al., 2005, 2009). Furthermore, reduced expression of UPS components in the spinal cord of SOD1G93A transgenic mice has been reported (Basso et al., 2009; Marino et al., 2015), and ALS mutant SOD1 itself is poly-ubiquitinated and cleared by the proteasome (Niwa et al., 2002; Urushitani et al., 2004). Possibly, age-related reductions in UPS activity or high demand, leads to the formation of cytotoxic mutant SOD1 inclusions (Kitamura et al., 2014).

Other familial ALS-associated proteins also support the role of a defective UPS in the development of ALS. In addition to its role in ERAD and ER stress discussed above, VCP is involved in substrate delivery to the proteasome (Dai and Li, 2001; Meyer and Weihl, 2014) and regulation of proteasome activity (Wojcik et al., 2004; Clemen et al., 2015). ALS-associated missense mutations in VCP have been shown to disrupt VCP-proteasome interaction, possibly resulting in defective proteasomal clearance of ubiquitinated proteins resulting in aggregation (Barthelme et al., 2015).

Mutations in *UBQLN2* which encodes the ubiquitin-like protein, ubiquilin-2, are associated with X-linked ALS and ALS-dementia (Deng et al., 2011). Ubiquilin-2 is a member of the ubiquilin family, which regulates the degradation of ubiquitinated proteins. Patients with ubiquilin-2 mutations display the classical ALS-associated ubiquitinated protein aggregates, which, interestingly, are also positive for ubiquilin-2 (Deng et al., 2011). Like VCP, ubiquilin-2 is able to bind poly-ubiquitin chains and aids in substrate delivery to the proteasome (Ko et al., 2004). ALS-associated ubiquilin-2 mutants are defective in proteasome binding, resulting in defective substrate delivery to the proteasome and the accumulation of poly-ubiquitinated proteasome substrates (Chang and Monteiro, 2015). Ubiquilin-2 has also been implicated in an autophagy independent pathway that clears protein aggregates via the proteasome. In this pathway, ubiquilin-2 shuttles Hsp70-bound aggregated proteins to the proteasome to allow degradation. ALS mutant ubiquilin-2 has lost the ability to bind to Hsp70 and this sensitizes cells to protein stress (Hjerpe et al., 2016).

As discussed above, ALS mutant VAPB is linked with ER stress and defective UPR. However, there is also evidence that ALS mutant VAPB may impair the UPS. VAPBP56S is found in cytoplasmic aggregates, which by itself could be an indication of defective proteasome function (Suzuki et al., 2009; Moumen et al., 2011). Expression of mutant VAPBP56S in cells promotes the formation of ubiquitinated aggregates, as well as the accumulation of other proteasomal substrates (Chen et al., 2010; Moumen et al., 2011). Similarly, ubiquitinated inclusions have been found in the spinal cord motor neurons of VAPBP56S transgenic mice (Tudor et al., 2010). Furthermore, mutant VAPBP56S has been demonstrated to interact with the 20S subunit of the proteasome, suggesting proteasome sequestering and trapping, and therefore UPS dysfunction, could be a contributing factor in ALS8 (Moumen et al., 2011).

Impaired proteasome function has also been reported in sporadic ALS cases (Kabashi et al., 2012). Levels of the 20S proteasome subunit were significantly reduced in motor neurons of sporadic ALS cases compared to controls, and proteasomal activity was found to be impaired in the spinal cord (Kabashi et al., 2012). As sporadic ALS cases make up the vast majority of clinically diagnosed ALS cases, this finding suggests that defective proteasomal activity may explain ALS pathology and be a contributing factor in a high percentage of ALS cases. In this context, it is noteworthy that conditional knockout of the proteasome subunit Rpt3 in motor neurons in mice results in motor neuron degeneration and locomotor defects accompanied by the accumulation of a number of ALS-related proteins, including TDP-43, FUS and optineurin in intraneuronal inclusions (Tashiro et al., 2012). Such inclusions are obviously reminiscent of the classical ALS pathology, suggesting that proteasomal dysfunction alone may be sufficient to drive the development of ALS.

### Autophagy

**FIGURE 6:**
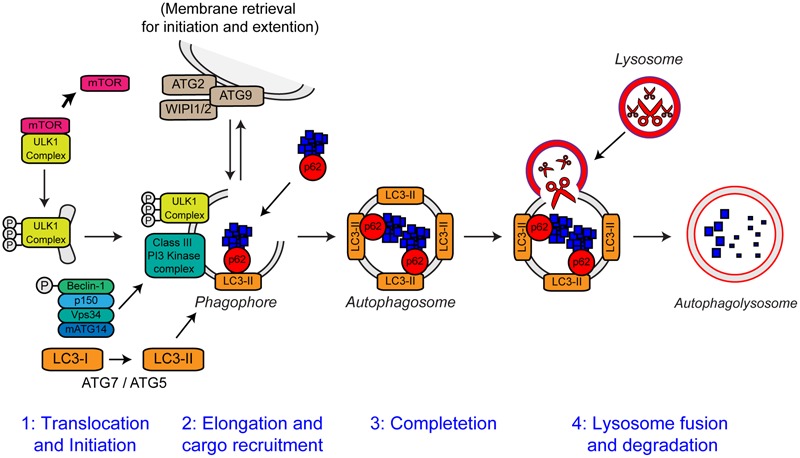
**The steps of autophagy.** The four stages of autophagy are indicated. (1) Translocation of the ULK1 initiation complex to the phagophore is the first step in autophagy initiation. Inhibition of mTOR, releases the ULK1 complex allowing activation and translocation of the complex. (2) Elongation of the phagophore membrane is mediated by the Class III PI3 kinase complex. Cargo recruitment to the growing phagophore is mediated by the autophagy receptors, p62/sequestosome-1 and optineurin. Autophagy receptors bind both poly-ubiquitin chains on autophagy substrates via the ubiquitin-like (Ubl) domains and LC3-II on the growing phagophore via LC3-interacting regions (LIRs). (3) After substrate recruitment and closure, completed autophagosomes are transported to allow fusion with the lysosome. (4) Autophagosome–lysosome fusion allows the degradation of the autophagic substrates by the action of acid hydrolases, present within the lysosome. Degradation allows the recycling of nutrients back to the cytosol. For further details please refer to the main text. Figure adapted from Webster et al. (2016b) under the terms of the Creative Commons Attribution License (CC BY).

Autophagy is an intracellular lysosomal degradation pathway responsible for the bulk clearance of cytoplasmic components such as misfolded proteins and damaged organelles. Autophagy requires the formation of a double membrane structure called the autophagosome, which encapsulates autophagic substrates prior to their transport to, and fusion with the lysosome (reviewed in Bento et al., 2016). The autophagic process can be divided into four distinct stages: (i) translocation and initiation, (ii) elongation and recruitment, (iii) completion, and (iv) lysosome fusion and degradation. The stages of autophagy are detailed in **Figure 6**.

Autophagy is essential for neuronal health. Inhibition of autophagy in neurons by neuronal-specific knockout of essential autophagy genes such as *Atg7, Atg5*, and *RB1CC1* (FIP200) causes neurodegeneration in mice in absence of any other contributory factors (Hara et al., 2006; Komatsu et al., 2006; Liang et al., 2010). Loss of autophagy in these mice is accompanied by progressive deficits in motor function, including abnormal limb-clasping reflexes (also observed in ALS mutant SOD1 transgenic mice) and a reduction in coordinated movement. Furthermore, reminiscent of many neurodegenerative diseases including ALS, loss of basal autophagy causes accumulation of neuronal ubiquitin-positive cytoplasmic inclusion bodies (Hara et al., 2006; Komatsu et al., 2006; Liang et al., 2010). Basal neuronal autophagy appears to be especially required for the maintenance of axons as loss of autophagy causes axonal dystrophy (Komatsu et al., 2006).

#### Defective Autophagy in ALS

Growing evidence supports a role of defective autophagy in the pathogenesis of ALS. As discussed above, ubiquitinated inclusion bodies are a neuropathological hallmark of ALS, suggesting autophagy may be compromised. Both SOD1 and TDP-43 are autophagy substrates, and compromised autophagy causes accumulation of mutant SOD1 and TDP-43 (Kabuta et al., 2006; Hetz et al., 2009; Wang et al., 2010; Brady et al., 2011; Barmada et al., 2014). Consistent with a block in autophagic flux, the levels of LC3-II are increased in SOD1G93A and H46R transgenic mice (Li et al., 2008; Hadano et al., 2010) as well as C9orf72 knockout mice (O’Rourke et al., 2016). However, it has to be noted that increased LC3-II levels may also be the result increased induction of autophagy in response to protein aggregation. Induction of autophagy using trehalose enhanced SOD1 clearance in NSC-34 cells and protected SOD1G93A transgenic mice (Castillo et al., 2013). Genetic induction of autophagy by XBP-1 knockout also extended lifespan in SOD1G93A transgenic mice (Hetz et al., 2009; Matus et al., 2009). Conversely, heterozygous deletion of Beclin-1 exacerbated disease in SOD1G127X transgenic mice (Tokuda et al., 2016). Autophagy enhancers increased TDP-43 turnover and prevented cell death in ALS mutant TDP-43 expressing cell lines but *in vivo* data are not yet available (Barmada et al., 2014).

There appears to be a regulatory feedback loop between TDP-43 and autophagy. TDP-43 has been shown to regulate the transcription of the essential autophagy gene *Atg7* (Bose et al., 2011). Loss of TDP-43 decreases Atg7 mRNA levels, in turn causing impairment of autophagy and accumulation of ubiquitinated proteins and p62/sequestosome-1. TDP-43, as well as FUS, regulate transcription of *HDAC6* which, along with its binding partner p62/sequestosome-1, plays an important role in the clearance of protein aggregates by aggrephagy (reviewed in Lamark and Johansen, 2012), such that loss of TDP-43 or FUS dramatically reduces the level of HDAC6 mRNA (Fiesel et al., 2010; Kim et al., 2010; Xia et al., 2015). Hence cytoplasmic aggregation of TDP-43/FUS, and associated loss of function, decreases *Atg7* and *HDAC6* expression and consequently autophagy while, conversely, reduced autophagy promotes TDP-43/FUS aggregation, in a perpetuating toxic loop.

#### The Function of ALS Genes in Autophagy

**FIGURE 7:**
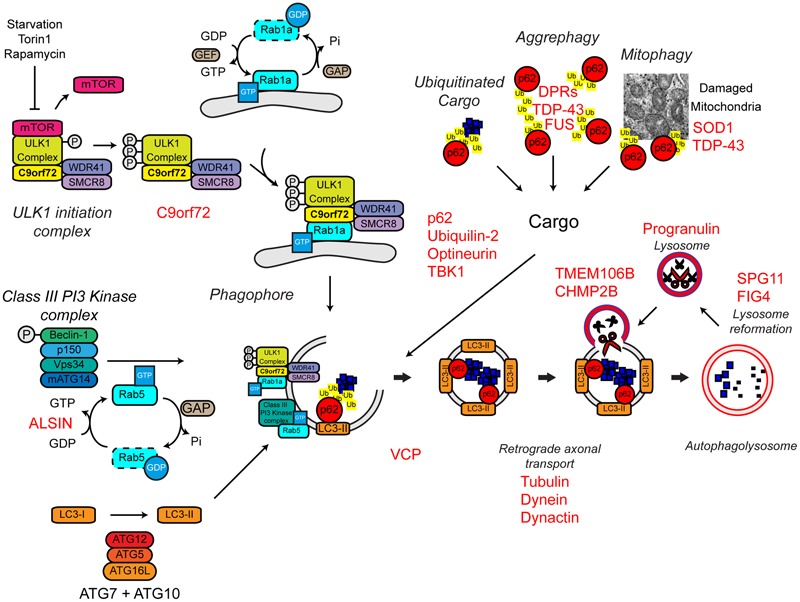
**Autophagy dysfunction and ALS.** Many ALS-associated genes, indicated in red, are implicated in the autophagy pathway. The location of gene names indicates the likely part of the pathway affected in ALS. Protein aggregates are a common feature of ALS pathology. The autophagy cargoes detailed here include a number of ALS-associated proteins, namely SOD1 and TDP-43, both of which are considered autophagy substrates. Mutant forms of these proteins, alterations to the pathway as a whole, or aberrant production of autophagy substrates, potentially in the case of C9orf72-related DPR proteins, may disrupt efficient substrate clearance, overwhelming the autophagy pathway and further promoting autophagy dysfunction. Importantly, TDP-43 is also important for autophagy gene transcription, thus participating as both a substrate and a regulator of the autophagy. For further details please refer to the main text. Figure adapted from Webster et al. (2016b) under the terms of the Creative Commons Attribution License (CC BY).

Several familial ALS genes function at different stages in the autophagy pathway (**Figure 7**).

##### Initiation

The most common genetic cause of ALS and FTD is a hexanucleotide repeat expansion of GGGGCC in the first intron of the *C9ORF72* gene (collectively termed C9ALS/FTD) (DeJesus-Hernandez et al., 2011; Renton et al., 2011). The pathogenic mechanisms behind this repeat expansion have been reviewed elsewhere, but may include toxic gain-of-function, namely via RNA toxicity and DPR formation, or alternatively loss-of-function via *C9ORF72* haploinsufficiency (reviewed in Cooper-Knock et al., 2015; Haeusler et al., 2016). With evidence for all three mechanisms in patients and a range of models, it is likely that all three mechanisms are at play. We and others have recently identified the C9orf72 protein as a regulator of autophagy (Amick et al., 2016; Sellier et al., 2016; Sullivan et al., 2016; Webster et al., 2016a; reviewed in Webster et al., 2016b; Yang et al., 2016). We found that C9orf72 is an effector of Rab1a that facilitates trafficking of the ULK1 initiation complex to the phagophore during autophagy initiation (Webster et al., 2016a) while others demonstrated that a complex of C9orf72, SMCR8, and WDR41 acts as a guanine nucleotide exchange factor for RAB8a and RAB39b further down the autophagy pathway during autophagosome maturation (Sellier et al., 2016). Furthermore, the C9orf72/SMCR8/WDR41 complex was shown to interact with the autophagy receptor optineurin (itself a Rab8a interactor) and TBK1, a well-known regulator of autophagy (Sellier et al., 2016). Both optineurin and TBK1 have been shown to be mutated in ALS cases (**Table 1**). Loss of C9orf72 in neurons inhibited autophagy and caused accumulation of p62/sequestosome-1 (Sellier et al., 2016; Webster et al., 2016a) while C9ALS/FTD patient-derived iNeurons exhibited reduced basal autophagy levels compared to controls (Webster et al., 2016a). Thus, these data predict that C9orf72 haploinsufficiency impairs autophagy. Consistent with compromised autophagy, C9ALS/FTD patients characteristically exhibit ubiquitin and p62/sequestosome-1 positive, TDP-43 negative, inclusions in the cerebellum and hippocampus (Al-Sarraj et al., 2011; Cooper-Knock et al., 2012; Mahoney et al., 2012).

In addition to C9orf72, another Rab GTPase-associated protein associated with autophagy is involved in ALS. Alsin is a GDP/GTP exchange factor for the small GTPase Rab5 (Topp et al., 2004). Rab5 is involved in autophagy initiation via activation of the Vps34 complex and the recruitment of the ATG12–ATG5 conjugation system required for autophagosome formation (Ravikumar et al., 2008; **Figure 7**). Pathogenic missense mutations in *ALS2* lead to loss of Alsin function, reduced Vps34 activation, defective autophagosome formation, and ultimately the development of ALS (Hadano et al., 2001, 2010; Otomo et al., 2011). Consistent with a role in autophagosome formation, genetic ablation of Alsin in SOD1H46R transgenic mice exacerbated disease and enhanced accumulation of a range of autophagy substrates including mutant SOD1 aggregates, poly-ubiquitinated proteins, and p62/sequestosome-1 (Hadano et al., 2010).

##### Delivery of substrates to the autophagosome

Autophagy receptors deliver cargoes to the forming autophagosome by binding to both ubiquitinated substrates and LC3-II via ubiquitin-associated (UBA) and LC3-interacting region (LIR) domains, respectively. Autophagy receptors are crucial for effective substrate clearance (reviewed in Stolz et al., 2014). Mutations in the autophagy receptors, ubiquilin-2, optineurin, and p62/sequestosome-1 have been shown to cause ALS (Fecto et al., 2011; Hirano et al., 2013; Teyssou et al., 2013; Chen et al., 2014; Majcher et al., 2015; **Figure 7**). These mutations appear to be loss-of function mutations that impair delivery of substrates to the autophagosome.

Mutations in ubiquilin-2, which have been found in both sporadic and familial ALS, lead to the incorporation of ubiquilin-2 into cytoplasmic aggregates in the brain and spinal cord (Deng et al., 2011). These aggregates are also positive for other ALS-related proteins, including TDP-43 and ubiquitin. Furthermore, ubiquilin-2 has been shown to interact directly with TDP-43 (Cassel and Reitz, 2013), suggesting that the mislocalisation of TDP-43 and its incorporation into cytoplasmic aggregates could be a product of defective autophagic clearance. Wild type ubiquilin-2 co-localizes with optineurin on p62/sequestosome-1 and ULK1 positive vesicles during autophagy induction (Osaka et al., 2015). However, the ALS-associated mutant ubiquilin-2 is absent from these optineurin positive vesicles, indicating a potential loss of function mechanism, and supporting the idea that ALS-associated mutations in ubiquilin-2 cause autophagic clearance defects (Osaka et al., 2015).

ALS-associated mutations within the *OPTN* gene lead to the increased formation of cytoplasmic aggregates, which are immunoreactive for optineurin itself (Maruyama et al., 2010; Ito et al., 2011; Korac et al., 2013; Weishaupt et al., 2013). The E478G mutation is found within the ubiquitin binding domain of optineurin, suggesting that, at least in the case of this mutant, substrate binding and therefore autophagosomal delivery may be compromised (Maruyama et al., 2010). It has also been reported that mutant optineurin can sequester and “trap” wild type proteins leading to defective autophagosome maturation, and therefore defective clearance (Shen et al., 2015).

ALS-associated mutations in p62/sequestosome-1 map to the LIR domain (Chen et al., 2014). One of these mutations, L341V, was shown to disrupt interaction with LC3-II (Goode et al., 2016), supporting the idea that p62/sequestosome-1 mutations disturb the delivery of autophagic substrates to the autophagosome. However, the effect of other mutations on the function of p62/sequestosome-1 and their effects on autophagy are yet to be determined.

The activities of p62/sequestosome-1 and optineurin are, in part, regulated by TBK1. Phosphorylation by TBK1 increases interaction of p62/sequestosome-1 and optineurin with LC3-II and/or ubiquitin chains, and increases autophagic clearance (Weidberg and Elazar, 2011; Wild et al., 2011; Matsumoto et al., 2015; Richter et al., 2016). As mentioned above, TBK1 also interacts with the C9orf72/SMCR8/WDR41 complex and phosphorylates SMCR8, but the significance of this phosphorylation is not yet clear (Sellier et al., 2016). Haploinsufficiency of TBK1 has been shown to cause ALS, likely by compromising its regulatory function in autophagy (Freischmidt et al., 2015).

##### Maturation

As previously discussed, VCP mutations have been found in both sporadic and familial ALS (**Table 1**). Although, VCP was initially identified as being involved in proteasome substrate delivery – see above (Dai and Li, 2001), there is also evidence supporting a role in autophagy. Reduced VCP activity leads to impaired maturation of autophagosomes and thus accumulation of ubiquitin and p62/sequestosome-1-positive aggregates (Ju et al., 2009; Tresse et al., 2010). VCP is also linked to two other ALS-associated proteins, TDP-43 and FUS. Both TDP-43 and FUS are involved in stress granule formation, cytoplasmic sites of stalled mRNA translation that form in response to stress (Buchan and Parker, 2009; Colombrita et al., 2009; Dormann et al., 2010; Bentmann et al., 2012). Mutant TDP-43 and FUS readily accumulate into stress granules (Bosco et al., 2010; Baron et al., 2013; Vance et al., 2013; Walker et al., 2013) and VCP and autophagy have been linked to stress granule clearance (Buchan et al., 2013).

FIG4 regulates the cellular levels of phosphatidylinositol-3,5-bisphosphate (PIP_2_), a signaling lipid required in autophagy (Ferguson et al., 2009). *FIG4* mutations lead to ALS potentially by a loss of function mechanism (Chow et al., 2009). *FIG4* knockout in mice leads to the accumulation of p62/sequestosome-1 and LC3-II, suggesting reduced autophagosome clearance could be at play in *FIG4*-related ALS (Ferguson et al., 2009).

Dynactin, a multimeric protein complex, interacts with cytoplasmic dynein to bring about retrograde transport of cargos (Waterman-Storer et al., 1997). Multiple point mutations in the p150 subunit of dynactin (*DCTN1*; dynactin-1) are found in a number of neurodegenerative diseases, including ALS (Puls et al., 2003; Münch et al., 2004, 2005; Vilariño-Güell et al., 2009). The ALS-associated mutations in dynactin may disrupt dynein–dynactin interactions, leading to aggregation of mutant dynactin and its respective cargos (Levy et al., 2006). Autophagosome transport in neurons is regulated by the dynein–dynactin complex and therefore disruption of this complex may lead to aberrant autophagosome transport and protein aggregation (Ravikumar et al., 2005; Maday et al., 2012; Maday and Holzbaur, 2014). In support of this, knock down of dynactin-1 in *Caenorhabditis elegans* causes motor defects, axonal degeneration and impaired transport and subsequent accumulation of autophagosomes (Ikenaka et al., 2013). Furthermore, reduced expression of dynactin-1 has been reported in sporadic ALS, suggesting alterations to the dynactin complex and disrupted autophagosome transport could be a common pathogenic mechanism in ALS (Jiang et al., 2007; Kuzma-Kozakiewicz et al., 2013).

##### Degradation

Missense mutations in the *charged multivesicular body protein 2B* (*CHMP2B*) gene is associated with FTD and predominantly lower motor neuron ALS (Skibinski et al., 2005; Parkinson et al., 2006; Cox et al., 2010). The CHMP2B protein is part of the endosomal sorting complex required for transport-III (ESCRT-III) (Bodon et al., 2011), which sorts ubiquitinated protein substrates from endosomes to the lysosomes by the formation of multivesicular bodies (MVBs) (reviewed in Williams and Urbe, 2007). Efficient autophagic degradation requires the formation of functional MVBs (Filimonenko et al., 2007). Interestingly, loss of ESCRT-III members or expression of mutant CHMP2B leads to the accumulation of autophagosome markers such as p62/sequestosome-1 and LC3-II consistent with reduced lysosomal clearance (Filimonenko et al., 2007; Lee et al., 2007; Urwin et al., 2010). Indeed impaired fusion events between the autophagosome and the lysosome has been demonstrated in HEK293 cells overexpressing mutant CHMP2B (Urwin et al., 2010).

Further evidence of defective autophagosome–lysosome fusion and clearance comes from the study of patients harboring mutations in *SPG11*, which encodes the Spatacsin protein. Deletions and frameshift mutations in *SPG11*, lead to hereditary spastic paraplegia (HSP) as well as juvenile recessive ALS (Orlacchio et al., 2010). Autophagosome accumulation in mutant *SPG11* patient fibroblast cells indicates that loss of spatacsin results in reduced clearance, potentially due to reduced lysosome biogenesis (Chang et al., 2014; Renvoisé et al., 2014). Data from mutant *SPG11* patient-derived iPSC neurons also indicates that loss of spatacsin may lead to impaired axonal transport, suggesting a convergence of pathogenic mechanisms (Pérez-Brangulí et al., 2014).

## Modulating Proteostasis to Treat ALS

The evidence discussed above strongly suggests that protein aggregation due to collapse of proteostasis may contribute to the etiology of ALS. Thus, restoring proteostasis to reduce aggregated protein burden has emerged as an attractive therapeutic target. Several pathways in the proteostasis network are amenable to pharmaceutical intervention and have been targeted in experimental models. To date, treatments have focused on either increasing protein folding to prevent protein aggregation or removal of aggregated proteins (**Table 2**).

**Table 2:** Restoring protein homeostasis as a treatment for ALS.

Target	Treatment	Effect on proteostasis	Effect on disease	Reference
HSF-1	Arimoclomol	Stabilizes HSF-1. Upregulation of chaperones	Delays disease progression and increases lifespan in SOD1G93A mice	Kieran et al., 2004; Kalmar et al., 2008
Hsp90	17-AAG	Hsp90 inhibition and Hsp70 activation	Reduces TDP-43 aggregates in HEK293 cells	Chang et al., 2013
UPR/eIF2α	Salubrinal	eIF2a maintained in active state and persistence of UPR	Protection of SOD1G93A mice MN from ER stress	Boyce et al., 2005
UPR/eIF2α	Guanabenz	eIF2a maintained in active state and persistence of UPR	Delayed disease onset, extended lifespan and reduced MN loss of SOD1G93A mice	Tsaytler et al., 2011
Chronic UPR	*XBP-1* knockout	Inhibition of UPR. Increased autophagic clearance	Extends survival in SOD1G86R mice and reduced SOD1 aggregation	Hetz et al., 2009
Chronic UPR	*ATF4* knockout	Complete inhibition of UPR pro-apoptotic gene expression	Delayed disease onset and extends survival in SOD1 G86R mice. Increased SOD1 aggregation	Matus et al., 2013
UPS	Pyrazolone	Proteasome activation	Reduced SOD1G93A cytotoxicity and aggregation	Chen et al., 2012; Trippier et al., 2012; Zhang et al., 2013
Autophagy	Fluphenazine methotrimeprazineNCP	Autophagy induction	Enhance mutant TDP-43A315T clearance, improved survival of murine primary cortical neurons overexpressing TDP-43A315T	Barmada et al., 2014
Autophagy	Berberine	Autophagy induction	Increased clearance of aggregate prone TDP-43 fragments in N2a cells	Chang et al., 2016
Autophagy	Rapamycin/tamoxifen	mTOR dependent autophagy induction	Reduces TDP-43 aggregation, rescues memory/learning and slow motor deficits in FTLD-U mouse	Wang et al., 2012
Autophagy	Spermidine/carbamazepine	mTOR independent autophagy induction	Reduced TDP-43 aggregation, increased memory/learning and reduced slow motor deficits in FTLD-U mouse	Wang et al., 2012
Autophagy	Trehalose	mTOR independent autophagy induction	Reduced SOD1G85R aggregation in spinal cord of SOD1G85R mice, delaying onset and increasing lifespan	Castillo et al., 2013; Zhang X. et al., 2014; Li et al., 2015

*MN, motor neurons.*

### Upregulation of Chaperone Function

As discussed above, molecular chaperones play an essential role in the proteostasis network. They are upregulated in response to protein stress (e.g., by the UPR), and thus are amenable to regulation. Furthermore, evidence suggests that inhibition of chaperone activity may contribute to ALS.

Hydroxylamine derivatives, such as Arimoclomol stabilize the transcription factor heat shock factor protein 1 (HSF-1) in its active state leading to its prolonged activation and the upregulation of a number of heat shock family chaperones, including Hsp60, Hsp70, and Hsp90 (Vígh et al., 1997). Arimoclomol has been shown to delay disease progression, extend lifespan, increase muscle function, and prevent aggregation of mutant SOD1 in SOD1G93A mouse models of ALS (Kieran et al., 2004; Kalmar et al., 2008, 2012). Importantly, HSF-1 does not bind its corresponding DNA elements in the absence of cellular stress. Thus, the prolonged activation of HSF-1 by hydroxylamine derivatives only enhances the heat shock response in already stressed cells, therefore mitigating the potentially toxic induction of the heat shock response and chaperone upregulation in otherwise healthy cell populations (Hargitai et al., 2003).

In a similar fashion to Arimoclomol, treatment with the Hsp90 inhibitor 17-AAG activates Hsp70 reducing the number of pathological TDP-43 aggregates in HEK293T cells overexpressing aggregation-prone TDP-43 C-terminal fragments (Chang et al., 2013).

### Treatments Targeting the UPR

ER stress appears to be involved in ALS pathogenesis. Accordingly, the UPR which alleviates ER stress under physiological conditions may be an attractive therapeutic target.

The PERK branch of the UPR leads to phosphorylation of the eukaryotic translational initiation factor eIF2α which decreases global protein synthesis and up-regulates the translation of selected stress-induced mRNAs such as the transcription factor ATF4. ATF4 induces expression of genes involved in amino acid metabolism, resistance to oxidative stress, and the proapoptotic transcription factor CHOP (**Figure 3**). The ER stress inhibitors salubrinal and guanabenz prevent eIF2α dephosphorylation and keep eIF2α in its inactive state (Boyce et al., 2005; Tsaytler et al., 2011). Salubrinal treatment protected SOD1G93A transgenic mouse motor neurons from ER stress (Saxena et al., 2009) and treatment of SOD1G93A transgenic mice with guanabenz delayed onset of disease, attenuated motor neuron loss and significantly extended lifespan (Jiang et al., 2014; Wang et al., 2014). In contrast, others have shown that guanabenz treatment accelerated disease progression in SOD1G93A transgenic mice (Vieira et al., 2015). The reason for these opposing findings is not clear, but may be attributable to differences in study design (discussed in Vieira et al., 2015). Both guanabenz and salubrinal attenuated ER stress and reduced paralysis and neurodegeneration in mutant TDP-43 *Caenorhabditis elegans* and zebrafish models of ALS (Vieira et al., 2015).

Perhaps counter-intuitively, conditional knockout of *XBP-1* was shown to extend the survival of female SOD1G86R transgenic mice (Hetz et al., 2009). However, in this model, abrogation of the UPR coincided with increased autophagic clearance of SOD1 aggregates, suggesting that a lower aggregated protein burden elicits the protective effect of *XBP-1* knockout (Hetz et al., 2009). Similarly, knockout of *ATF4* also delayed disease onset and prolonged life span in SOD1G86R transgenic mice. ATF4 deficiency completely prevented the induction of pro-apoptotic genes, but increased SOD1 aggregation suggesting that, in this case, prevention of UPR-induced apoptosis mediated protection (Matus et al., 2013).

### Increasing Degradation of Misfolded Proteins and Aggregates

While increasing protein folding may help to reduce the pathological protein accumulations in ALS, an alternative approach is to promote their degradation by activation of the UPS and/or autophagy.

#### Proteasome Activation

Pyrazolone-containing small molecules have been shown to block ALS mutant SOD1 mediated cytotoxicity and aggregation in a number of studies (Chen et al., 2012; Trippier et al., 2012; Zhang et al., 2013). The mechanism of action of pyrazolone was shown to be by activation of the proteasome, suggesting that proteasome activators may be of therapeutic benefit in ALS (Trippier et al., 2014). However, the protective action of pyrazolones has only been tested in mutant SOD1-related ALS models, and as such more studies are needed to test the efficacy of these small molecules in other ALS models.

#### Enhancing Autophagy

Most attempts to modulate autophagy as a treatment for ALS have been aimed at enhancing the removal of aggregated proteins by stimulating autophagic flux. *In vitro*, three potent inducers of autophagy, fluphenazine, methotrimeprazine and 10-(4′-(*N*-diethylamino)butyl)-2-chlorophenoxazine, efficiently enhance ALS mutant TDP-43A315T aggregate clearance and improved survival of murine primary cortical neurons overexpressing TDP-43A315T as well as iPSC-derived motor neurons and astrocytes carrying the pathogenic M337V TDP-43 mutation (Barmada et al., 2014). Similarly, the traditional medicinal herb and known inducer of autophagy, berberine, increased clearance of EGFP-tagged aggregation-prone C-terminal TDP-43 fragments, and reduced insoluble TDP-43 aggregates in transfected Neuro2a cells (Chang et al., 2016). *In vivo*, a number of mTOR-dependent (rapamycin, tamoxifen) and independent (spermidine, carbamazepine) autophagy enhancers have been shown to reduce TDP-43 aggregation, rescue learning/memory and slow motor deficits in an FTLD-U mouse model with transgenic overexpression of TDP-43 in the hippocampus, cortex, and striatum (Wang et al., 2012). Hence induction of autophagy may be of therapeutic benefit to clear ALS-associated TDP-43 aggregates.

Activating autophagy may also be beneficial in the removal of mutant SOD1 aggregates. Trehalose, an mTOR independent activator of autophagy, has been shown to reduce the aggregation of SOD1G85R in the spinal cord of SOD1G85R transgenic mice, an effect that was accompanied by increased lifespan and delayed disease onset in these mice (Castillo et al., 2013). Similar results were reported in SOD1G93A transgenic mice (Zhang X. et al., 2014), although the efficacy of trehalose may decline as the disease progresses (Li et al., 2015). In contrast to trehalose, mTOR dependent induction of autophagy using rapamycin has either no effect (Staats et al., 2013) or exacerbates disease in SOD1G93A transgenic mice (Zhang et al., 2011). This effect of rapamycin may stem from its function as an immunosuppressant, because rapamycin treatment did increase survival in SOD1G93A transgenic mice lacking mature lymphocytes (Staats et al., 2013).

## Discussion and Outlook

Collapse of proteostasis and resulting protein aggregation is a universal feature of ALS as discussed above. Most evidence suggest that protein aggregation is deleterious for cells and, by extension, this may also be the case in ALS. In agreement, boosting clearance of protein aggregates and/or preventing protein aggregation appears to be of benefit in animal models of ALS. However, it has been suggested that protein aggregation may be a protective mechanism. By compartmentalization of the toxic misfolded proteins the cell prevents them from causing damage, e.g., to mitochondria or to proteostasis by sequestering chaperones and blocking the UPS. Furthermore, compartmentalization may facilitate clearance by aggrephagy. Thus, some caution is warranted when devising treatments to prevent protein aggregation.

Proteostasis is a universal process in every cell, not just neurons. Before embarking on long term treatment with for example autophagy enhancers, possible side effects need to be considered. For example, evidence from the cancer field indicates that in most cases autophagy facilitates tumorigenesis (reviewed in White, 2015). Similarly, inhibition of the PERK branch of the UPR may seem a good idea in principle but balancing the UPR appears to be critical for neuronal integrity, and long-term PERK inhibition may not be feasible due to considerable side effects (Scheper and Hoozemans, 2013). Furthermore, is important to take into account the genetic background of individual ALS patients when considering employing modulators of protein quality control as a treatment of disease. For example, treating patients with a known autophagosome clearance defect with autophagy enhancers would not be likely to be beneficial and may even be deleterious.

Nevertheless, results from ALS models and models of other neurodegenerative diseases such as Huntington’s disease indicate that treatments targeting proteostasis and in particular autophagy have great potential. Further understanding of the genetics of ALS and the mechanisms of proteostasis dysfunction associated with ALS will be vital if proteostasis modulators are to be used effectively as neuroprotective therapies.

## Author Contributions

CW, ES, and KDV: wrote the manuscript. CW: prepared the figures. PS and KDV: edited the manuscript and figures.

## Conflict of Interest Statement

The authors declare that the research was conducted in the absence of any commercial or financial relationships that could be construed as a potential conflict of interest.
